# Evaluation of the impact of delayed centrifugation on the diagnostic performance of serum creatinine as a baseline measure of renal function before antiretroviral treatment

**DOI:** 10.4102/sajhivmed.v21i1.1056

**Published:** 2020-07-16

**Authors:** Chemedzai E. Chikomba, Carolyn J. Padoa, Donald Tanyanyiwa

**Affiliations:** 1 Department of Chemical Pathology, National Health Laboratory Services, Faculty of Health Science, University of the Witwatersrand, Johannesburg, South Africa; 2 Department of Chemical Pathology, National Health Laboratory Service, Faculty of Health Science, Sefako Makgatho Health Sciences University, Pretoria, South Africa

**Keywords:** Kidney function, serum creatinine, antiretroviral, estimated GFR, kinetic Jaffe, i-STAT, ROCHE

## Abstract

**Background:**

The measurement of serum creatinine is a standard requirement of the medical management of people living with HIV. Renal dysfunction is common, both as a complication of HIV-infection and as a result of its treatment. The detection of abnormal renal function before the start of antiretroviral therapy will impact patient management and the outcome of treatment.

**Objectives:**

This study aimed to determine if a time delay in the centrifugation of serum samples affected the creatinine level and the estimated glomerular filtration rate as recorded on the analytical platforms used in the laboratory.

**Methods:**

Twenty-two (*n* = 22) HIV-positive, newly diagnosed and treatment-naïve patients were randomly recruited from Alexandra Health Clinic, Johannesburg, South Africa. Serum samples were centrifuged at six time intervals following receipt of the sample viz. < 4 h (baseline), 6 h, 24 h, 48 h, 72 h and 96 h. Creatinine concentrations were measured on the Roche platform utilising the enzymatic and kinetic Jaffe methods. Whole blood samples were also analysed with the Abbott i-STAT point-of-care instrument. The estimated glomerular filtration rate was calculated using the Cockcroft Gault, CKD–Epidemiology Collaboration and Modified Diet and Renal Disease v3/4 equations.

**Results:**

At baseline (< 4 h) there was good agreement between the enzymatic and kinetic Jaffe methods: bias 1.7 µmol/l. The enzymatic and i-STAT creatinine concentrations were stable over 96 h viz. changes of 1.8% and 5.7%. However, from 24 h onwards agreement between the enzymatic and kinetic Jaffe methods was poor with the latter measuring 43.7 µmol/l higher than the enzymatic method at 96 h. Creatinine concentrations measured with the kinetic Jaffe method increased significantly in samples centrifuged after 6 h (*p* < 0.001, 61.7% change), and resulted in a 95% decline in eGFR at 96 h as determined with the CKD–Epidemiology Collaboration equation.

**Conclusion:**

The analysis of serum creatinine using the isotope dilution mass spectrometry traceable kinetic Jaffe method is unreliable if performed on samples centrifuged ≥ 6 h after collection. The raised creatinine concentration can affect clinical decisions such as renal functional assessment, choice of antiretroviral drug or regimen, and the dose and frequency of medication.

## Introduction

South Africa (SA) has one of the highest HIV infection rates in the world. In 2016, the overall prevalence of HIV infection in SA was 12.7%.^1,2^ The use of antiretroviral drugs (ARVs) has slowed the progression of the infection and increased the life expectancy of people living with HIV.^3,4,5^ In SA’s public health sector, patients are started on antiretroviral treatment (ART) with combinations of different ARV drug classes, prescribed as a single, once-a-day tablet.^6^ This has led to a reduction of the pill-burden of therapy and to improved adherence.^7,8^ In the southern African region the most frequently prescribed initial combination is the **nucleotide** reverse transcriptase inhibitor (NtRTI), tenofovir disoproxil fumarate (TDF), the **nucleoside** reverse transcriptase inhibitors (NRTIs) emtricitabine (FTC) or lamivudine (3TC) and the non-nucleoside reverse transcriptase inhibitor (NNRTI), efavirenz (EFV). This is first-line ART.^6^ Current SA national ART guidelines indicate that the integrase strand transfer inhibitor (INSTI), dolutegravir (DTG), has replaced efavirenz for all except women of child-bearing age who may fall pregnant or those initiating ART in the first trimester of pregnancy.^9,10^ Tenofovir however remains the standard NRTI-backbone of all first-line ART in southern Africa.

TDF is an acyclic nucleoside phosphate prodrug. Its long half-life permits once-daily dosing. Although the drug is extensively filtered by the kidneys, 20% – 30% is actively reabsorbed at the proximal tubule.^11^ TDF is an occasional cause of renal injury viz. a proximal renal tubulopathy and a salt-wasting syndrome (Fanconi Syndrome and renal tubular acidosis), drug-induced acute kidney injury and a slower, general decline in glomerular filtration, diabetes insipidus, and a dysregulation of the kidney’s calcium and phosphorus metabolism.^11,12^ Impaired renal function may also result from the use of other drugs, such as aminoglycosides, sulphonamides, and amphotericin B, the presence of HIV-associated nephropathy (HIVAN), blood-borne infection, for example tuberculosis, bacteraemia and fungaemia, HIV-associated immune-complex kidney disease and common comorbidities such as hypertension and diabetes mellitus.^13,14^ Pre-existing mild renal dysfunction may increase susceptibility to the toxicity of TDF. Kidney function must be evaluated before starting ART: the use of TDF is contraindicated if the estimated glomerular filtration rate (eGFR) is < 50 mL/min. Patients on TDF have serum creatinine measured at 3 and 6 months following the initiation of ART and biannually thereafter. In high-risk patients, for example with coexistent hypertension or diabetes, the creatinine is measured more frequently.^9^

The measurement of the GFR confirms and stages the degree of renal impairment and provides a platform for the ongoing monitoring of kidney function. Although the urinary clearance of inulin is the gold standard of GFR measurement,^15^ the method is time-consuming, expensive and impractical in most clinical settings. Endogenous substances such as serum creatinine and cystatin C are cheaper, provide more rapid results and are widely available.^16^ Although the National Institute for Health and Care Excellence (NICE) and the Kidney Disease Improving Global Outcomes (KDIGO) groups both base their eGFR assessment (equations) on cystatin C, its cost and lack of standardisation excludes its general use.^17,18^ Creatinine is produced at a constant rate, is present in all body fluids, and is filtered by the glomerulus. Its serum level is influenced by several biological factors, such as active secretion by the renal tubules in the presence of declining renal function, diet, extremes of muscle mass, age, gender, drugs and the use of creatine supplements.^19^ Several equations control for some of these variables.^20^ The SA National Department of Health (SANDOH) 2019 guidelines recommend therefore that the Counahan-Barratt equation be used to measure the eGFR of youths aged 10–16 years and the Modifications of Diet in Renal Disease (MDRD) equation be used for adolescents and adults 16 years of age and older.^9^

The Cockcroft-Gault (CG) equation incorporates the serum creatinine level and the variables of weight, age and sex in the measurement of the eGFR.^21^ The equation has limitations: in pregnancy, at the extremes of weight, and in those on dialysis for acute renal failure. Its principal use is in drug dosing and pharmacokinetic studies.^20^ The MDRD equation adds a fourth variable: race.^22^ This ethnicity adjustment-factor is based on African Americans and tends to overestimate the GFR in sub-Saharan (African) populations.^23^ The CKD–Epidemiology Collaboration (EPI) equation is recommended by the KDIGO guidelines group and has been validated in participants with and without impaired renal function with eGFR < 90 mL/min/1.73 min².^17,24^ The SA National Health Laboratory Service (SA-NHLS) currently reports both the MDRD and CKD-EPI eGFR without ethnic adjustment. The adoption of the CKD-EPI equation in SA has been delayed due to limited local data.

Routine laboratory serum creatinine measurements are analysed on Jaffe and enzymatic assays. These methods should be traceable to isotope dilution mass spectrometry (IDMS) method and the Standard Reference Material for creatinine in serum (SRM 967). NHLS laboratories in SA generally use the modified kinetic Jaffe method, as opposed to the enzymatic assay, as it is more affordable. The World Health Organization’s (WHO) guidelines indicate that at room temperature, creatinine is stable for 2–3 days.^25^ However, a report from Shepherd et al.^26^ noted a significant increase in creatinine levels measured on the kinetic Jaffe method when processing occurred later than 24 h.^26^ This impacted the study’s eGFR results and had caused the renal misclassification of patients.^26^ A turnaround time for serum creatinine measurements from local clinics in Gauteng of 12–72 h has been observed at NHLS laboratories, as reported in the internal TAT reports. This delay increases with the remoteness of clinics due to the poor transport networks, the restricted working hours at referral laboratories, and the greater workload of regional laboratories. The aim of this study was to determine, in HIV-positive black South Africans not on ART, the stability limit (SL), that is, the time at room temperature, when the serum creatinine results are still within the maximum permissible instability (MPI) range.^27^

## Methods

### Study population

Twenty-two (*n* = 22) newly diagnosed and treatment-naïve people living with HIV between the ages of 18 and 70 years were randomly recruited from the Alexandra Health Community Centre in Alexandra, Johannesburg, SA. The minimum number of participants required for testing the stability of biochemical analytes is 10.^27^ Furthermore, multiple samples were required from each participant for analysis on three analysers at six time points, providing 396 creatinine measurement data points. Patients with comorbidities such as diabetes mellitus, pregnancy, urinary tract infection, hypertension and proteinuria were excluded from the study. De-identified demographic and anthropometric data were transcribed from the patient files.

### Sample collection and processing

Each patient provided 13 tubes of blood: 12 × approximately 2 mL in 5 mL serum separator-tubes (SST) and 1 × 5 mL in a heparin tube (Becton Dickenson, Plymouth, UK). Serum was isolated from SST tubes following centrifugation at 1370 × g for 5 min at defined time intervals viz. < 4 h, 6 h, 24 h, 48 h, 72 h and 96 h. Prior to centrifugation, samples were stored at room temperature. Whole blood heparin tubes were stored at room temperature until required.

### Measurement of creatinine concentrations

Serum creatinine concentrations were measured immediately after each centrifugation (as above) using the IDMS-traceable enzymatic and IDMS-traceable kinetic Jaffe method on the Roche COBAS 8000 module 702 and Roche COBAS 6000 module 502 instruments at the NHLS laboratory of the Chris Hani Baragwanath Hospital, Soweto, SA. The enzymatic method test principle is based on a series of coupled reactions that result in the conversion of creatinine to hydrogen peroxide. In the final reaction, catalysed by peroxidase, a quinone imine chromogen is formed. The colour intensity of this chromogen is measured at 550 nm and is directly proportional to the concentration of creatinine in the sample. The kinetic Jaffe method test principle is based on the reaction of creatinine and picrate under alkaline conditions forming a yellow-orange complex. The rate of formation of this complex is proportional to the amount of creatinine in the sample. The kinetic Jaffe method is prone to interference from non-creatinine chromogens such as proteins and ketones. A correction factor of -26 µmol/L is, therefore, universally applied to correct for these inherent chromogens. Bilirubin is the major negative interferent in the kinetic Jaffe method, and this is overcome by rate blanking. The internal quality control for creatinine in our laboratory is performed twice daily. The laboratory adheres to the Royal College of Pathologists of Australasia (RCPA) quality assurance programmes. External quality assurance submissions during the study period were within allowable limits.

Creatinine concentrations were also measured using whole blood samples on the Abbott i-STAT point-of-care instrument immediately after collection and at 6 h, 24 h, 48 h, 72 h and 96 h. This method is based on the conversion of creatinine to hydrogen peroxide, via hydrolysis and oxidation. The hydrogen peroxide is oxidized (at the platinum electrode) to produce a current that is proportional to the concentration of creatinine in the sample.

### Estimated glomerular filtration rate measurements

eGFR was calculated using the MDRDv3 *without ethnic adjustment* (age, sex, and serum creatinine), MDRDv4 (age, sex, ethnicity, and serum creatinine), CG and CKD-EPI equations *with ethnic adjustment.*

### Statistical analysis

All analyses were performed using Medcalc software. The Shapiro-Wilks test was used to test for normality. Normally distributed continuous variables (creatinine) were presented as mean ± standard deviation and continuous variables that were not normally distributed (age, weight) were presented as median [interquartile range]. Categorical variables are presented as proportions and percentages. The Student paired t-test was used to compare baseline creatinine concentrations between the three different methods (Bonferroni corrected *p*-value). The serum creatinine results were evaluated for analytically significant changes using the uncertainty of measurement (UOM; calculated for the laboratory quality control data in the preceding 6 months), total allowable error (TAE) of 15% (Clinical Laboratory Improvement Amendments [CLIA] and coefficient of variation [CV]). Passing-Bablok linear regression and Bland-Altman plots were generated to evaluate correlation and method agreement over time, using the enzymatic method as the reference method. For all analyses, *p* < 0.05 was considered statistically significant.

### Ethical consideration

Ethical approval was obtained from the University of the Witwatersrand Human Research Ethic Committee, reference number: M1711109. R14/49.

## Results

### Characteristics of the study population

A total of 22 randomly recruited HIV-positive, treatment-naïve black South African individuals consented to participate in this study. The median age of the participants was 37 years. The majority (54.5%; 12 of 22) of participants were younger than 40 years of age. The age distribution is consistent with the current demographics published by Statistics South Africa with respect to individuals mostly affected by HIV/AIDS. All participants were black Africans (ethnicity self-reported). The median weight for the participants was 70.0 kg (range 45–110 kg) and 59.1% (13 of 22) of participants were male.

### Creatinine assay performance

The mean baseline (< 4 h) creatinine concentrations for the study cohort obtained using the enzymatic, kinetic Jaffe method and i-STAT methods were 78.73 µmol/L ±14.63 µmol/L, 77.05 µmol/L ± 13.12 µmol/L and 70.14 µmol/L ± 15.3 µmol/L: *p* > 0.05 for all comparisons (Table 1). The mean creatinine concentrations analysed using the kinetic Jaffe method were significantly higher than baseline when blood samples were processed after 6 h: *p* ≤ 0.001 for all comparisons. Creatinine concentrations did not change significantly over the 96 h when measured using the enzymatic method or i-STAT system: *p* > 0.05 for all comparisons (Table 1).

**TABLE 1:** Creatinine concentrations for the study cohort (*n* = 22) obtained using the enzymatic, kinetic Jaffe method and i-STAT methods at six different time intervals.

Time interval (hours)	Creatinine concentration					
	Enzymatic		Kinetic Jaffe		i-STAT	
	µmol/l	*p	µmol/L	*p	µmol/L	*p
< 4 h	78.73 ± 14.63	-	77.05 ± 13.12	-	70.14 ± 15.35	-
6 h	74.55 ± 14.11	0.340	78.41 ± 15.19	0.752	69.91 ± 16.00	0.962
24 h	78.91 ± 14.64	0.967	95.23 ± 20.26	0.001	67.59 ± 14.72	0.578
48 h	79.86 ± 15.70	0.805	117.64 ± 19.82	< 0.001	68.18 ± 14.02	0.662
72 h	76.79 ± 14.82†	0.676	121.29 ± 20.97§	< 0.001	67.59 ± 14.71	0.577
96 h	77.31 ± 10.45‡	0.743	124.56 ± 18.49¶	< 0.001	66.14 ± 15.82††	0.406

Results are expressed as mean ± standard deviation, missing data due to insufficient volume or high haemolysis index (> 1000 mg/dL) for

† , *n* = 3.

‡ , *n* = 6.

§ , *n* = 1.

¶ , *n* = 4.

†† , *n* =1.

* , *p*-value for results compared to 4 h creatinine concentration.

**FIGURE 1:**
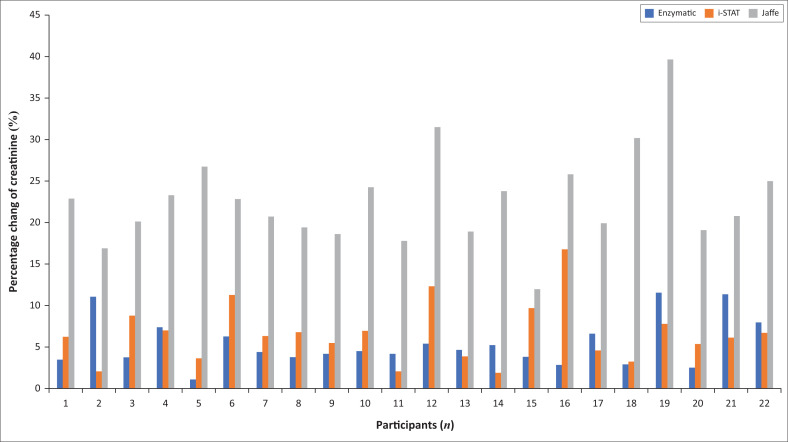
Intra-individual coefficient of variation for 22 participants for the enzymatic, kinetic Jaffe and i-STAT creatinine methods over the 96-h study period. Blue bars represent results obtained for the enzymatic method, orange bars for the i-STAT method and grey bars for the kinetic Jaffe method.

The kinetic Jaffe method exhibited the widest intra-individual CV (range: 12% – 40% vs 1.2% – 12% enzymatic and 1.9% – 12% i-STAT method) as well as the greatest inter-assay variation (22.7% vs 6.0% enzymatic and 6.6% i-STAT method), as seen in Figure 1. When creatinine results were assessed against the 10% UOM for each respective assay, 8.9% of the enzymatic creatinine results fell outside the UOM for the duration of the study compared to 65% of the results for the kinetic Jaffe method. The i-STAT results could not be evaluated as UOM has not been established for this assay.

**FIGURE 2:**
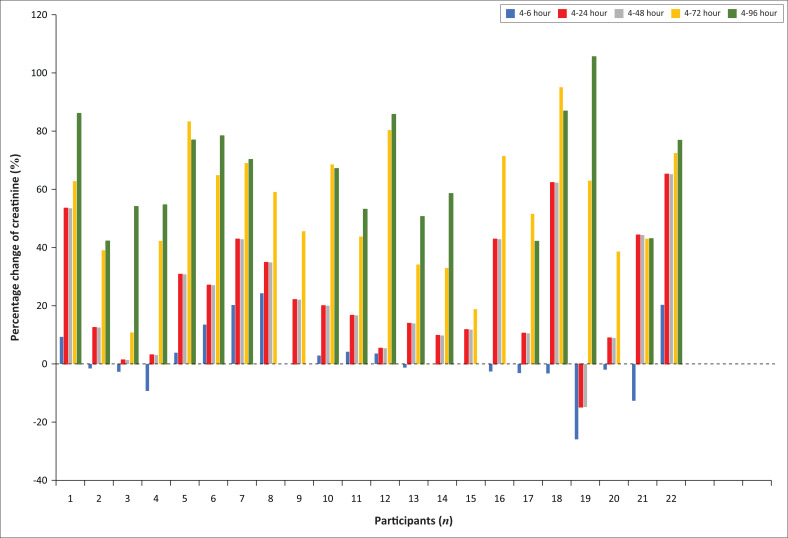
Percentage change in serum creatinine concentrations obtained using the kinetic Jaffe method over 96 h for 22 participants relative to the serum creatinine results at 4 h. Percentage change to baseline (4 h): blue bars = 4–6 h, orange = 4–24 h, grey = 4–48 h, yellow = 4–72 h, green = 4–96 h.

Based on the positive trend of increasing creatinine concentrations over time observed for the Jaffe method, we explored the data further to determine if a correction factor could be established to adjust creatinine results obtained from samples with delayed processing time. The percentage change of the serum creatinine results for the participants over the 96-h time frame was inconsistent and a correction factor could, therefore, not be established based on the current sample size (Figure 2).

### Method comparison

**FIGURE 3:**
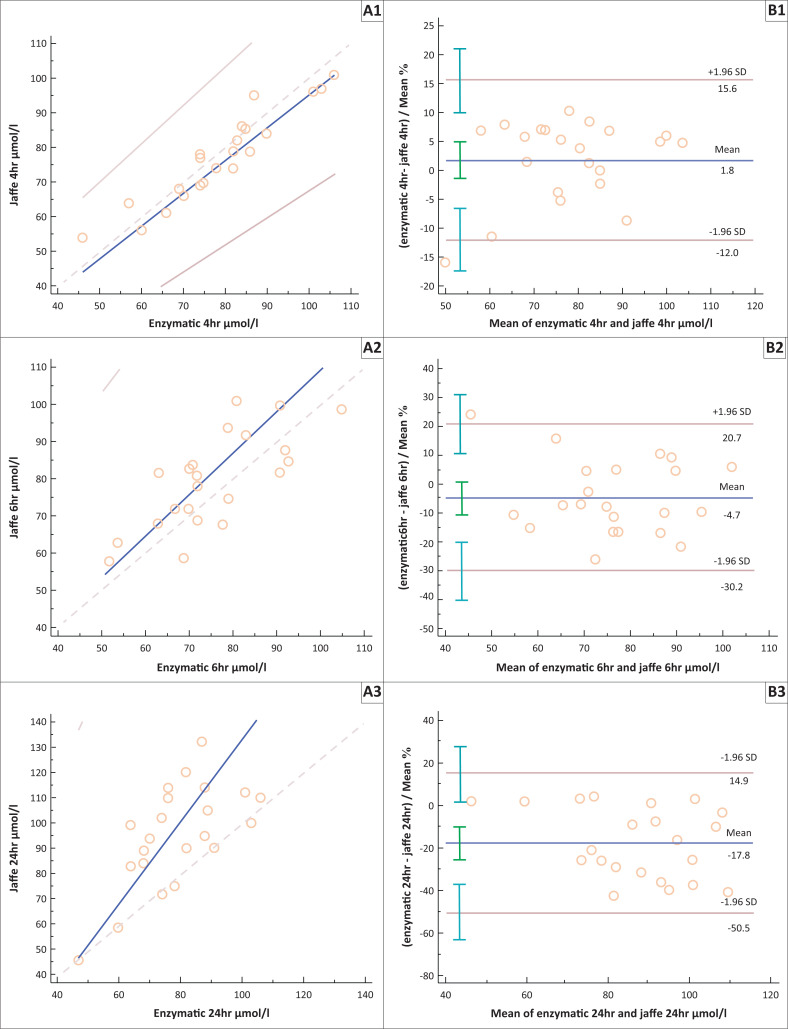

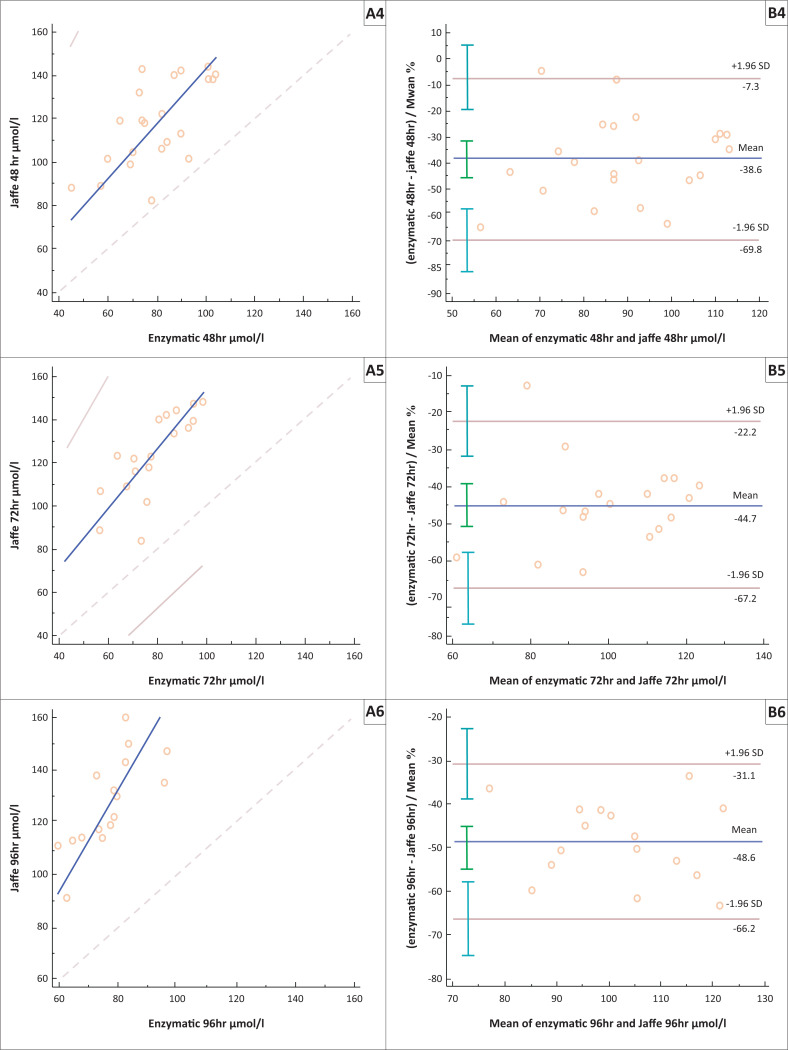
Passing-Bablok regression curves (A) and Bland-Altman plots (B) comparing creatinine concentrations from the kinetic Jaffe method with those from the enzymatic method. Creatinine concentrations at (1) < 4 h, (2) 6 h, (3) 24 h, (4) 48 h, (5) 72 h and (6) 96 h.

**FIGURE 4:**
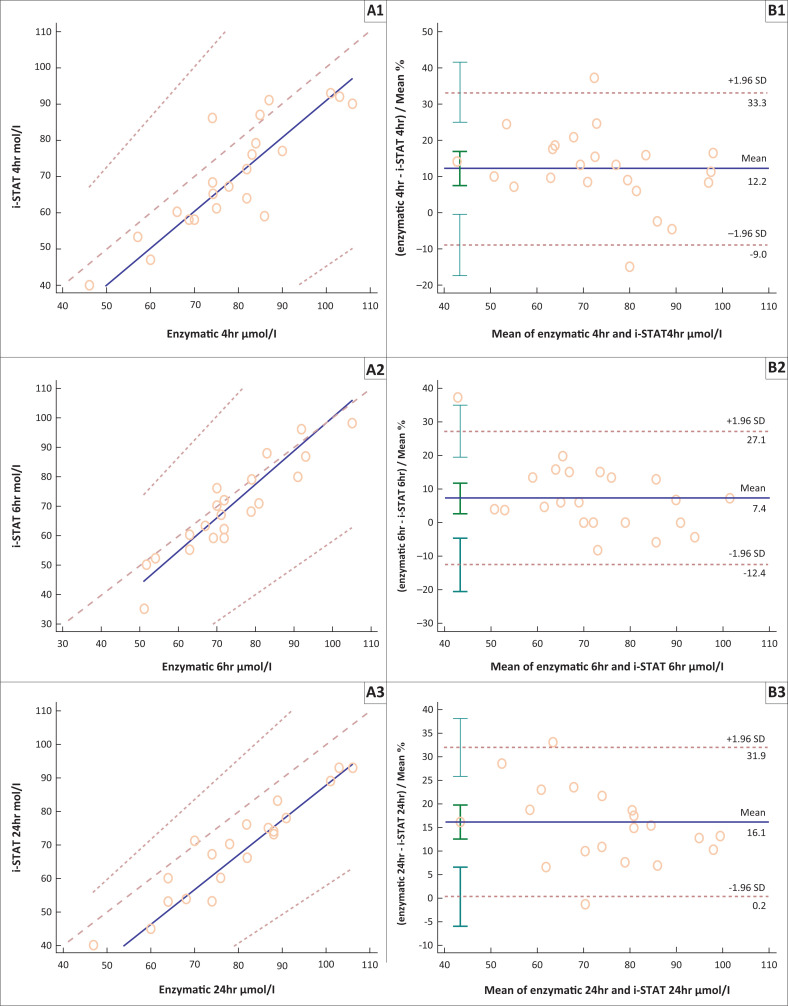

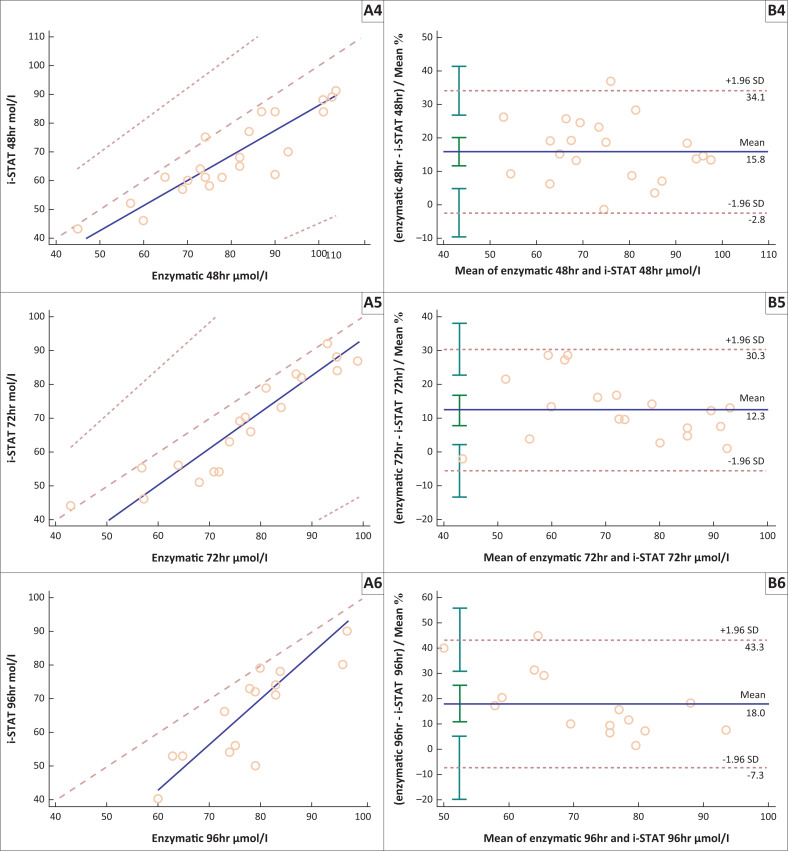
Passing-Bablok regression curves (A) and Bland-Altman plots (B) comparing creatinine concentrations from the i-STAT device with those obtained from the enzymatic method. Creatinine concentrations at (1) < 4 h, (2) 6 h, (3) 24 h, (4) 48 h, (5) 72 h and (6) 96 h.

Passing-Bablok regression and Bland-Altman plots were used to determine the correlation and agreement of the kinetic Jaffe method and i-STAT creatinine results compared to the enzymatic creatinine results at different time intervals (see Table 2, Figure 3 and Figure 4). The enzymatic method was chosen as the reference method as it had the best analytical CV in our study and previous studies have shown that this method correlated well with the standard reference method (isotopic dilution mass spectrometry).^27,28,29^

**TABLE 2:** Comparison of serum creatinine levels from kinetic Jaffe method and i-STAT method to the enzymatic method using Passing-Bablok and Bland-Altman analysis.

Analytical method	Time (hours)	Passing–Bablok regression parameters				Bland-Altman			
		Slope	95% CI	Intercept (95% CI)	95% CI	*r*	Mean bias	%	Mean bias, µmol/l
Kinetic Jaffe Method	< 4 h	0.95	0.78–1.1	0.54	−10.74–15.54	0.953	1.8	−1.31–4.93	1.7
	6 h	1.10	0.75–1.64	−2.00	−41.18–21.25	0.803	−4.7	−10.47–1.04	3.9
	24 h	1.6	0.95–2.50	−27.83	−88.50–19.65	0.596	−17.8	−25.20–10.40	16.3
	48 h	1.26	0.83–2.23	16.84	−56.85–52.75	0.582	−38.6	−45.66–-31.51	37.8
	72 h	1.33	1.06–2.00	18.90	−32.00–40.13	0.858†	−44.7	−50.24–39.18	44.0
	96 h	2.25	1.13–5.50	−23.82	152.40–43.94	0.855‡	−48.6	−53.41–43.88	49.9
i-STAT	< 4 h	1.01	0.83–1.38	−10.53	−38.23–3.50	0.836	12.2	7.38–16.96	8.6
	6 h	1.13	0.91–1.40	−13.24	−33.00–2.50	0.900	7.4	2.89–11.84	4.6
	24 h	1.04	0.86–1.20	−16.30	−28.50–−0.59	0.923	16.0	12.48–19.65	11.7
	48 h	0.88	0.67–1.12	−1.35	−21.54–13.67	0.893	15.8	11.69–19.95	11.6
	72 h	1.08	0.86–1.37	−14.61	−38.41–2.57	0.948§	12.3	7.93–16.74	8.6
	96 h	1.35	1.00–2.00	−38.02	−90.00–10.00	0.847¶	18.0	10.86–25.16	12.0

Legend: *r* = correlation coefficient, linear regression line *y* = *mx* + *c, c* = intercept (constant error), *m* = slope (proportional error), number of participants results analysed.

† , *n* = 19.

‡ , *n* = 16.

§ , *n* = 19.

¶ , *n* = 16.

The Jaffe and enzymatic method showed a strong correlation at < 4 h (*r* = 0.953); however, this correlation became weaker with time. The higher *r* values at 72 h and 96 h may be due to the missing creatinine data (enzymatic 72 h *n* = 3, enzymatic 96 h *n* = 6, Jaffe 72 h *n* = 1, Jaffe 96 h *n* = 4) for participants at these time intervals. The slope and the intercept increased over the time interval (Table 2), and this demonstrated the increase in the magnitude of the systematic error. At 4 h, there was a strong agreement between the kinetic Jaffe method and enzymatic methods with a small negative bias of 1.8% (1.7 µmol/L). With an increased delay in sample separation, the kinetic Jaffe method resulted in an overestimation of creatinine concentrations with a positive bias of 48.6% (49.9 µmol/l) at 96 h (Figure 3).

Creatinine results from the i-STAT correlated well with the enzymatic creatinine results over the six time intervals used in the study: *r*-value, 0.836–0.948. The i-STAT method had a negative bias ranging from 7.4% to 18.0% throughout the study. The i-STAT method had a negative bias of 12.2% (8.6 µmol/L) at 4 h and 18.0% (12.0 µmol/L) at 96 h.

### Comparison of estimated glomerular filtration rate equation performance

The impact on the classification of renal dysfunction of the delay in centrifugation of blood and serum samples measured for creatinine levels by means of three different laboratory methsods was evaluated with four eGFR equations viz. CG, MDRD v4, MDRD v3, and CKD-EPI. The serum creatinine eGFR results of the enzymatic and i-STAT methods performed well (to within the 10% TAE for eGFR throughout the study). However, the eGFR from the Jaffe data decreased over time. At baseline, viz. < 4 h, there was consensus among all four equations. All 22 participants had an eGFR > 60 mL/min per 1.73 m² using the CG and MDRD v4 equation. Similarly, 21 participants had an eGFR > 60 mL/min per 1.73 m² with the MDRD v3 and the CKD-EPI equations (Table 3). One participant had an eGFR of < 60 mL/min per 1.73 m². This person was classified as having stage 3A renal failure with an eGFR 45 mL/min – 59 mL/min per 1.73 m², according to the KDIGO guidelines.

**TABLE 3:** Chronic Kidney Disease staging using the four equations based on creatinine results obtained from the enzymatic, kinetic Jaffe and i-STAT methods.

Measure	Enzymatic								Kinetic Jaffe								i-STAT							
	4 h		24 h		96 h		4 h		24 h		96 h		4 h		24 h		96 h	
	*n*	%	*n*	%	*n*	%	*n*	%	*n*	%	*n*	%	*n*	%	*n*	%	*n*	%
**CKD-EPI**																		
eGFR mean (mL/min per 1.73 m²)	118 ± 36		118 ± 37		114 ± 28		100 ± 26		75± 33		45 ± 10		147 ± 50		152 ± 56		161 ± 68	
Range	53–217		53–210		73–148		48–150		34–181		24–63		63–269		62–269		81–269	
15–29	0	0	0	0	0	0	0	0	0	0	1	5.5	0	0	0	0	0	0
30–44	0	0	0	0	0	0	0	0	2	9	9	50	0	0	0	0	0	0
45–59	1	4.5	1	4.5	0	0	1	0.5	6	27	7	39	0	0	0	0	0	0
60–89	5	23	4	18	5	31	7	32	10	45	1	5.5	2	9	2	9	2	10
≥ 90	16	73	17	77	11	69	14	64	4	18	0	0	20	91	20	91	19	90
**MDRDv3**																		
eGFR mean (mL/min per 1.73 m²)	89 ± 20		89 ± 21		89 ± 15		91 ± 18		83 ± 31		51 ± 9		104 ± 126		107 ± 27		112 ± 34	
Range	49–141		40–137		62–108		52–125		55–190		31–67		55–166		55–166		68–166	
15–29	0	0	0	0	0	0	0	0	0	0	0	0	0	0	0	0	0	0
30–44	0	0	0	0	0	0	0	0	0	0	4	22	0	0	0	0	0	0
45–59	1	4.5	2	9	0	0	1	4.5	3	14	12	67	1	4.5	1	4.5	0	0
> 60	21	95.5	20	91	16	100	21	95.5	19	86	2	11	21	95.5	21	95.5	21	100
**MDRDv4**																		
eGFR mean (mL/min per 1.73 m²)	108 ± 25		108 ± 26		108 ± 18		100 ± 22		69 ± 26		42 ± 7		125 ± 32		130 ± 32		136 ± 41	
Range	59–171		59–166		75–133		63–151		45–157		26–55		67–200		66–209		82–201	
15–29	0	0	0	0	0	0	0	0	0	0	1	5.5	0	0	0	0	0	0
30–44	0	0	0	0	0	0	0	0	0	0	10	5.5	0	0	0	0	0	0
45–59	1	4.5	2	9	0	0	0	0	11	9	7	39	0	0	1	4.5	0	0
> 60	21	95.5	20	91	16	100	22	100	11	8	0	0	22	100	21	-	21	100
**CG**																		
eGFR mean (mL/min per 1.73 m²)	129 ± 66		128 ± 64		125 ± 51		129 ± 54		111 ± 69		79 ± 28		125 ± 52		130 ± 51		137 ± 57	
Range	66–391		66–382		79–295		69–333		54–391		47–162		78–328		76–324		83–324	
15–29	0	0	0	0	0	0	0	0	0	0	0	0	0	0	0	0	0	0
30–44	0	0	0	0	0	0	0	0	0	0	0	0	0	0	0	0	0	0
45–59	0	0	0	0	0	0	0	0	1	4.5	2	11	0	0	0	0	0	0
> 60	22	100	22	100	16	100	22	100	21	95.5	16	89	22	100	22	100	21	100

CKD-EPI, Chronic Kidney Disease-Epidemiology; eGFR, estimated glomerular filtration rate; MDRD, Modifications of Diet in Renal Disease; CG, Cockcroft-Gault.

**FIGURE 5:**
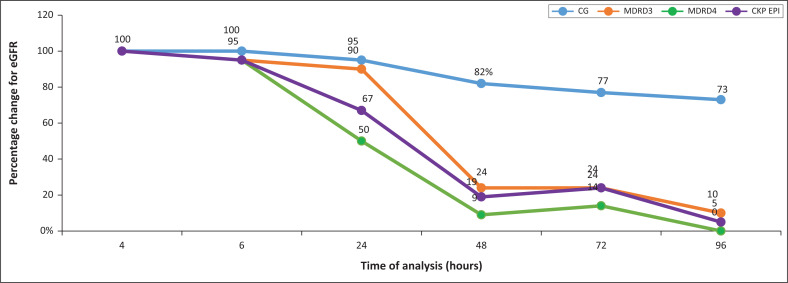
Percentage change of estimated glomerular filtration rate classification based on kinetic Jaffe creatinine concentrations over the 96-h study period compared to estimated glomerular filtration rate obtained within 4 h using the Cockcroft-Gault, Modifications of Diet in Renal Disease v3, Modifications of Diet in Renal Disease v4, and Chronic Kidney Disease-Epidemiology equations. blue = CG, orange = MDRD3, purple = CKD, green = MDRD4.

The change in eGFR varied over time among the different equations. The CG equation was the least sensitive to the increase in serum creatinine: only 27% (*n* = 6/22) of eGFR-based 96-h creatinine concentrations resulted in a change in the Chronic Kidney Disease (CKD) stage when compared with baseline (i.e. the < 4-h eGFR; see Figure 5). The MDRD v4 equation gave the greatest change in eGFR over 96 h: 50% (*n* = 11/22) of participants at 24 h and 100% (*n* = 22/22) at 96 h showed an eGFR decline compared to baseline. The most significant change of eGFR was noted with the MDRDv3 equation: 25% eGFR at 48 h and 10% at 96 h compared to the baseline eGFR.

**FIGURE 6:**
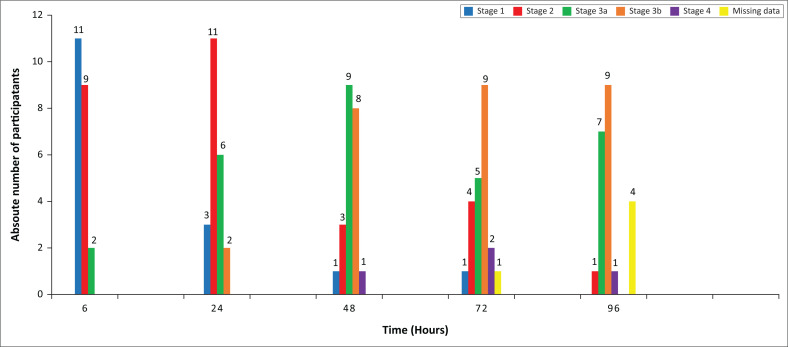
The number of participants classified per renal stage at each time interval using the serum creatinine results obtained from the kinetic Jaffe method to calculate estimated glomerular filtration rate using the Chronic Kidney Disease-Epidemiology equation. Blue bars = stage 1 (eGFR ≥ 90 mL/min per 1.73 m²), Red bars = stage 2 (eGFR 60 mL/min per 1.73 m² – 89 mL/min per 1.73 m²), Green bars = stage 3a (eGFR 45 mL/min per 1.73 m² – 59 mL/min per 1.73 m²), Orange bars = stage 3b (eGFR 30 mL/min per 1.73 m² – 44 mL/min per 1.73 m²), Purple bars = stage 4 (15 eGFR mL/min per 1.73 m²– 29 eGFR mL/min per 1.73 m²), Missing data due to haemolysis or insufficient sample = yellow bars.

Using the CKD-EPI equation as recommended by the KDIGO guidelines, enzymatic and i-STAT renal classification was noted at different time intervals with changes between stages 1 and 2, which are not clinically significant for ARV drug choice. Using the kinetic Jaffe method however, changes in the renal classification were observed for 21 participants over the 96-h study period. At baseline, < 4 h, 21 of 22 (95%) participants had an eGFR > 60 mL/min/1.73 m² (renal stage 1 and 2) while at 96 h only 1 of 18 (6%) participant was classified as stage 2. The remaining 17 participants (94%) were classified as stage 3a or higher (eGFR < 60 mL/min/1.73 m²). Figure 6 demonstrates that renal staging deteriorated over the study period, with all participants (100%) eligible for the tenofovir based regimen with an eGFR > 50 mL/min/1.73 m² at 4 h while only four participants (22%) would have been eligible for this regimen at 96 h.

## Discussion

The accurate measurement of serum and whole-blood creatinine is essential to the determination of the eGFR. Laboratory methods have been standardised, but concern remains regarding the impact on results of nonspecific analytical biases.^30,31^ This study from a clinical laboratory based at a large public hospital in SA reports levels of serum creatinine as assessed by three standard methods of measurement, following a delay in sample separation and centrifugation. The clinical significance of the delay was evaluated by calculating the eGFR of each of the 22 patient’s serum samples by means of the following four equations: the CG, MDRD v4, MDRD v3, and CKD-EPI. Serum and whole blood creatinine as assessed by the enzymatic and i-STAT methods were stable throughout the study. However, we found that a delay in centrifugation and sample separation of > 6 h resulted in significantly raised creatinine concentrations when using the kinetic Jaffe method. This led to the misclassification of patients when the eGFR was determined using all four formulas (equations), and was consistent with previous studies.^29,32^

The Roche enzymatic creatinine results were stable over the duration of the study, with a CV of 6%, which was consistent with a previous study which showed that the enzymatic method meets the analytical performance specifications as stipulated by the National Kidney Disease Education Program (NKDEP) (USA).^33,34^ The i-STAT method showed a mean negative bias of 13.6 µmol/L compared to the enzymatic method. Studies comparing the performance of the i-STAT device to other platforms have been inconsistent.^35,36,37,38^ One study showed that the i-STAT overestimated creatinine results by 3.88 µmol/L in comparison to the Roche enzymatic creatinine results. This overestimation occurred predominantly at higher creatinine concentrations. In contrast, a study conducted on oncology patients found that mean creatinine concentrations obtained on the i-STAT system were 42.4 µmol/lower than those obtained using the core laboratory method.^35^ The negative bias may be attributed to whole blood creatinine negative interferences.^39^ POCT creatinine whole blood samples are affected by the matrix effect (such as haematocrit) and are prone to negative bias.^40^

Despite the negative bias seen with the i-STAT device, the creatinine results were sufficiently sensitive to detect the participant with renal dysfunction using the MDRD v3 and CKD-EPI equations. There are currently no POCT analytical goals for creatinine and no UOM was available as this was a new platform. However, the negative bias and the CV were within the acceptable ‘Laboratory Working Group of the NKDEP’ total error goal of less than 10% for the eGFR (CV < 8% and an analytical bias relative to IDMS < 5%).^41^ Previous studies have cautioned utilisation of POCT creatinine due to failure to detect renal dysfunction; however, in our study the i-STAT did not erroneously classify any of the participants.^42^ It is worth noting that the equations for eGFR were derived using serum samples, therefore utilisation of these equations with whole blood samples should still be validated using measured GFR.

While standardisation of creatinine methods has addressed calibration biases, method-specific interferences remain problematic.^30^ In the current study, the Roche kinetic Jaffe method showed analytical, clinically significant imprecision and a positive bias for creatinine results when analysed 24 h after collection. This finding is consistent with previous studies that showed delayed separation of serum from cellular components following specimen collection resulted in a positive interference with different Jaffe methods and could be attributed to the accumulation of pyruvate and other non-creatinine chromogens.^26^ Ford et al.^32^ established that on the Roche kinetic Jaffe method the serum creatinine results were significantly increased from 16 h and recommended that creatinine results with a delay in separation should not be reported as they impacted CKD staging. Similarly, Shepherd et al.^26^showed an overestimation of Jaffe creatinine results when assessing the impact of delayed sample separation using five different analytical platforms.^26^ These results are in contrast to the WHO guidelines that state that creatinine in whole blood is stable at room temperature for 2–3 days.^25^ In the current study, the overestimation by the kinetic Jaffe method over time was clinically significant as this led to a higher CKD staging (i.e. lower eGFR). This is consistent with a study by Drion et al. where they found that creatinine results from Jaffe techniques were more biased, imprecise and overestimated serum creatinine concentrations, especially at low concentrations compared to enzymatic techniques.^29^ The authors recommended that clinical laboratories consider the health costs associated with inappropriate referral and further suggested using the enzymatic assay that had a minimal bias.

The inconsistencies observed in renal classification using the four GFR equations shown in this study highlight the fact that these equations cannot be used interchangeably for identifying kidney disease, monitoring of renal function, drug dosing and selection of ARV drug regimens. Measured GFR was not required for this study as ascertaining the GFR was not pertinent, but rather the study sought to quantify the clinical significance of the impact of the instability on the calculated GFR. The CG equation was the least sensitive to the effect of the increased creatinine concentrations on eGFR in our study. This is consistent with a previous finding that demonstrated that the CG equation overestimated the eGFR in a large cohort of Europeans, resulting in the misclassification of 29.2% of individuals.^43^ In contrast, the CKD-EPI equation was sensitive to the serum creatinine changes of the kinetic Jaffe method, and the impact on the eGFR was noted at 24 h. This result is consistent with a previous study by Seape and colleagues who found that the CKD-EPI equation performed better than other creatinine-based eGFR equations when assessing renal function in 100 treatment-naïve HIV-positive individuals.^44^

## Strengths and limitations

This study was able to show the impact of delayed processing of samples on creatinine concentrations on three platforms with a larger sample size than previous studies. In addition, blood samples were taken from participants at a single time point and the pre-analytical conditions were standardised. A limitation of this study is that serum indices (such as bilirubin which can falsely elevate creatinine results) were not considered when evaluating the creatinine result. Furthermore, we did not exclude all confounders to creatinine measurements such as drugs and pre-existing renal dysfunction which may have adversely affected the creatinine results at all time intervals. In addition, we did not use the reference creatinine method (IDMS) for comparison purposes; we used an enzymatic method traceable to IDMS. A further limitation is that we did not have a wide range of creatinine concentrations to see how a delay in centrifugation would affect the higher concentrations. Furthermore, this study did not explore all the risks associated with POCT devices such as lot-to-lot variation (one lot number was used), operator variability, quality control, analytical traceability, impact of temperature, humidity, different sample types, and the different analytical performance of different POCT devices which have previously been shown to have a significant impact on the eGFR.^45^

## Conclusion

This study demonstrated that the Roche kinetic Jaffe method creatinine assay gave falsely elevated serum creatinine levels if samples were not separated and analysed within 24 h of collection. The increase in the serum creatinine concentration over time was clinically significant resulting in misclassification of renal status which would, in turn, lead to incorrect clinical decisions regarding ARV regimen choice. Based on the outputs of this study, and previously published literature,^26,44^ laboratories currently analysing creatinine on any Jaffe method should reject (not analyse) samples reaching the laboratory after the stability index time from collection (< 24 h), or if these laboratories decide to analyse these serum creatinine samples an appropriate interpretative comment should be appended to the result as done for other biochemical analytes such as potassium, glucose, and phosphate. The enzymatic creatinine method is highly recommended in clinical laboratories that analyse samples more than 24 h after collection due to transport delays and excessive workload. A major deterrent concerning the utility of the enzymatic method is its high cost. However, the health costs associated with the misclassification of patients using the Jaffe method may outweigh the analytical costs of the enzymatic method. Increased demand for the enzymatic method will result in competition between suppliers and ultimately reduce the cost. This study demonstrated that although creatinine assays have been standardised the extra analytical considerations which are not standardised may result in clinically significant creatinine results, thus the use of POCT, which performed well in this study, in remote clinics may avert the need to perform the laboratory-based kinetic Jaffe.

Measurement of creatinine with a POCT provides an accurate, timely result, and has eliminated the pre-analytical problems noted with the kinetic Jaffe method; therefore, adopting of POCT creatinine should be envisaged. The i-STAT POCT creatinine, however, had a negative bias which resulted in the misdiagnosis of a renal dysfunction for one participant with stage 3a renal failure while the eGFR of the other 21 participants was not affected. Its utilisation in HIV/AIDS programmes could, therefore, be adopted once the eGFR equations have been validated for the sample type, confounders of POCT mentioned previously and infrastructural issues such as integration with the laboratory information systems have been addressed. POCT and enzymatic assays will reduce unnecessary clinic visits due to renal misdiagnoses (false positives) and importantly reduce the pill burden, thereby ultimately reducing health costs to both the government and the patient.
